# Managing Aged Animals in Zoos to Promote Positive Welfare: A Review and Future Directions

**DOI:** 10.3390/ani8070116

**Published:** 2018-07-13

**Authors:** Bethany L. Krebs, Debra Marrin, Amy Phelps, Lana Krol, Jason V. Watters

**Affiliations:** San Francisco Zoological Society, San Francisco, CA 94132, USA; DebbieM@sfzoo.org (D.M.); AmyP@sfzoo.org (A.P.); LanaK@sfzoo.org (L.K.); JasonW@sfzoo.org (J.V.W.)

**Keywords:** geriatric, ageing, comparative gerontology, zoo biology

## Abstract

**Simple Summary:**

Many animals experience physical and behavioral changes as they age. Age-related changes in physical or mental ability can limit the opportunities for animals to experience positive well-being. As animals in zoos are living longer than ever, understanding common physical, cognitive, and behavioral changes associated with ageing across species can help inform management practices. This review aggregates information about common age-related changes across a wide number of species, discusses the potential welfare impacts of these changes for ageing animals, and suggests methods for caretakers to maximize positive welfare opportunities for ageing animals under human care.

**Abstract:**

Improvements in veterinary care, nutrition, and husbandry of animals living in zoos have led to an increase in the longevity of these animals over the past 30 years. In this same time period, the focus of animal welfare science has shifted from concerns over mitigating negative welfare impacts to promoting positive welfare experiences for animals. For instance, providing opportunities for animals to exert agency, solve problems, or acquire rewards are all associated with positive welfare outcomes. Many common age-related changes result in limitations to opportunities for positive welfare experiences, either due to pain or other physical, cognitive, or behavioral limitations. This review aggregates information regarding common age-related physical and behavioral changes across species, discusses how age-related changes may limit positive welfare opportunities of aged animals in human care, and suggests potential management methods to help promote positive welfare for animals at all life stages in zoos and aquariums.

## 1. Introduction

Zoological facilities care for a vast array of species, for which there may be limited longevity data, veterinary reference values, behavioral baselines, or even standardized care practices. Recognizing this, accrediting organizations are developing animal care manuals that collate as much information about a species as possible to support their care. For example, the Association of Zoos and Aquariums (AZA) now has 27 animal care manuals published and 25 currently in progress. With modern veterinary medicine and nutrition science, many animals that are cared for by humans now survive well past the average lifespan of their wild counterparts [1]. Ensuring a positive quality of life during these years is a growing concern among those who care for animals [2]. The challenges of caring for ageing animals are manifold and information regarding age-related changes in many species living in zoos is limited. The increasing focus on ensuring positive well-being for animals in accredited zoos and aquariums across all life stages, coupled with the increase in overall longevity of the species living in zoos and aquariums highlights the current need for a clearer understanding of challenges and considerations involved in providing care and supporting positive welfare opportunities for aged animals.

Zoos and aquariums are increasingly focused on animal welfare. Early welfare work focused on eliminating animals’ negative experiences (e.g., freedom from pain [3]), however in recent years animal welfare science has increasingly focused on the positive well-being of animals [4]. Moreover, modern animal welfare science acknowledges providing proper environmental provisioning for an animal is necessary but not sufficient for ensuring the positive welfare of each individual of a given species [5,6]. Positive welfare is associated with numerous opportunities including the ability to exert agency in daily life [7,8,9], engagement with the environment (e.g., physical and social aspects of one’s world [10,11,12,13,14]), problem solving [9,15,16,17,18,19,20,21], and acquiring rewards [22]. As welfare is a state experienced by an individual and dependent upon one’s preferences, experiences, and personality, supporting positive welfare requires an understanding of characteristics that can impact welfare states. 

Much of the existing work on promoting positive welfare states assumes animals living in adequately provisioned conditions are free from negative welfare experiences. This assumption may be more true of animals in some age classes than others, as surveys of pain in aged human populations suggest >70% of elderly humans experience some form of physical pain regularly [23,24]. Necropsies of aged domestic pets as well as zoo animals indicate high prevalence of conditions at time of death likely to cause pain [2,25,26,27]. Thus, animals in different age classes may require different care or opportunities to support positive welfare, with aged animals potentially being at greater risk for negative welfare experiences such as pain or distress. In view of this, understanding common age-related challenges experienced by older animals under human care has important welfare implications for ageing zoo and aquarium populations.

Within accredited zoological organizations, ‘populations’ of zoo and aquarium animals consist of individual or small groups of animals that are housed at numerous different locations. Due to the geographic distribution of these populations as well as the relative rarity of individuals in the oldest age classes within any population [28], information about ageing in many exotic species is limited to individual case studies or may be a covariate in studies of other topics [29,30]. Many case studies are published due to the abnormal nature or outcome of the associated case, and may not represent the typical ageing process for a given species [31,32]; nonetheless, such cases help illustrate the diversity of species experiencing age-related changes. A further complication of caring for aged animals is that although many individuals of the same species may reach advanced age within the global population, any single facility may only care for one or two aged individuals of that species at any given time. Even when an institution has recently (e.g., in the past 20 years) cared for multiple aged individuals of a species, advances in management, nutrition, and veterinary care in the same time period can make it difficult to determine how best to care for a species in old age. This lack of clarity may occur particularly for long lived species that can experience many different management methods over their life spans [33,34]. As such, the information available about ageing in zoo or aquarium species historically may be of limited use for guiding the management of living animals in modern zoological institutions.

In addition to changes that occur as a result of the evolution of husbandry and care processes, the idiosyncrasy of ageing means even animals of the same species will experience different age-related changes at different times, and consequently require different care [2,35,36]. Nonetheless, some information available from zoological institutions, as well as livestock and small animal veterinary practices, suggest a number of common changes to health or behavior associated with age across species. The purpose of this review is to aggregate available information on age-related changes to health, cognition, and behavior, drawing on the available information from zoo case studies, ageing in humans, and the growing body of veterinary research on geriatric care for domestic animals and pets. We highlight age-related changes to physical health and behavior observed across taxa, and discuss potential welfare concerns related to the changes. From this information, we propose general principles for managing aged animals in zoos to promote positive welfare, and suggest areas where future research is needed to advance the care of older animals in zoos and aquariums.

## 2. Definition of Senescence or Ageing

The physical and psychological changes colloquially referred to as ‘ageing’ are more technically defined as senescence. Senescence is characterized by a suite of physiological changes leading to decline or cessation of reproduction and increased risk of mortality [37]. Senescent changes impact all systems of the body in a cumulative manner, eventually resulting in loss of function and death, however this process is not pathological in itself and normal ageing is not considered a medical diagnosis [38,39]. Advanced age is often a risk factor for the development of a variety of diseases, and so senescent changes often occur alongside or may contribute to the development of pathological processes [38]. Hereafter, the terms ‘ageing’ or ‘age-related’ will be used in the colloquial sense throughout the review, although certain changes described could occur either as part of normal senescence or due to pathological changes. Although it is not possible to mitigate the impacts of all senescent changes an animal experiences, the impact of such changes on the animal’s comfort level or psychological well-being may warrant non-medical interventions, which is the focus of this review. Determining the nature of changes an aged animal experiences (i.e., senescent or pathological) requires assessment by veterinary staff, and will benefit from input from caretakers, managers, and staff behaviorists. The management suggestions discussed here are presumed to be undertaken in conjunction with proper veterinary care and treatment, and are intended to be additional inputs for promoting comfort and positive welfare opportunities for animals of advanced age.

## 3. Physical Changes

### 3.1. Body Mass and Composition

Ageing in humans can be associated with changes in body composition—such as a decrease in lean body mass and increase in body fat—as well as overall declines in body weight [40,41]. Similar patterns have been reported in older domestic cats (*Felis catus*), dogs (*Canis lupus familiaris*), and horses (*Equus caballus*) [42,43,44,45]. Ageing humans and dogs may not exhibit weight loss with increasing age, but may still experience a loss of muscle mass and increase in body fat as body composition shifts with age [42,46,47]. Gradual declines in overall body mass may thus be associated with normal ageing in a number of taxa. Slow declines in body mass may be related to a decrease in resting metabolic rate associated with changes in body composition, a decline in overall caloric intake [44,48], age-related changes to digestive function or transit time [44], pain related inappetence [49], or hormonal changes [50,51]. Weight loss may also be attributable to any number of underlying medical issues [45,49,52], therefore sustained or unexpected changes to an animal’s body condition should always be assessed by a veterinarian for underlying causes. Older animals may also be at greater risk for becoming overweight or obese due to declining resting metabolic rates and overall lower activity levels if there is no corresponding decrease in caloric intake as activity levels decline [48]. Decreases in muscle mass may be associated with declines in mobility over time as well [44,53], highlighting the importance of maintaining age appropriate physical activity for older animals.

Maintaining physical activity is important at all life stages, however it may be more critical for elderly animals who are less active than younger ones [54]. Physical exercise has been shown to maintain lean body mass and muscle strength in elderly humans and dogs, and moderate physical activity at any life stage appears to have positive impacts on cognitive ability in ageing humans and in animal models [55]. Although many older animals will opt to become less active as they age [44,56,57], daily enrichment opportunities can be used to stimulate physical conditioning and movement. Enrichment may need to be modified for the animal’s changing abilities to maintain its effectiveness (e.g., an animal may no longer be able to easily climb up high to reach food hidden in elevated areas though it is still motivated to search for reward [58,59]). Positive reinforcement training can provide external motivation for physical activity, and stimulate interest in food in individuals with low appetite, as age-related declines in activity level may be directly related to physiological changes in the dopamine driven motivation system [60,61]. Training may also provide opportunities to engage animals in physical therapy exercises. To maximize the benefits of training, protocols may need to be adjusted for changes in an animals’ physical abilities. Routine assessment of engagement with enrichment and training opportunities can help identify enrichments or trained behaviors that need adjustments for the animal’s ability. Thoughtful applications of positive reinforcement training and enrichment opportunities can help maintain activity and thus animals’ body condition.

Changes in body composition associated with ageing can result in weight gain or weight loss [41,42]. Changes to animals’ diets should always be undertaken with the help of an animal nutritionist or qualified veterinarian, particularly when weight adjustment is the end goal. For animals experiencing changes in body mass, maintaining a variety of food items in the diet can be useful for achieving weight management goals. Animals showing decreased appetites or intakes can benefit from calorically dense supplemental feedings to help maintain body weight [62]. If weight changes are determined to be related to changes in digestion or transit times, animals may benefit from easier to digest alternative feeds or dietary supplements such as fiber [62,63,64,65,66,67,68,69]. Maintaining flexibility in an animal’s diet at younger life stages whenever possible can prepare the animal to be flexible about dietary changes or alternate foods should weight management issues arise as the animal ages.

Although body composition may seem to be a largely medical issue, weight management, enrichment, and training efforts undertaken for aged animals have welfare implications as well. Proper feeding and nutrition are critical to physical health of animals of all ages, and likewise maintaining animals at an appropriate body weight can mitigate health risks associated with being overweight at any life stage. Maintaining a proper body weight throughout an animal’s life can help avert the development of some conditions as the animal ages, and weight loss can help relieve mobility issues or symptoms of degenerative joint problems [25,49,51,70,71,72]. Weight loss is not always desirable, however, and decreased appetite and associated weight loss can be indicative of pain or disease [45,49]. Weight loss from pain-related inappetence is cause for welfare concerns for the individual, as it can indicate the animal has been living with pain for an extended period. Even for individuals who were considered overweight beforehand, pain related weight loss should not be considered a de facto ‘management success’. In humans, being overweight does not appear to have inherent negative impacts on subjective psychological well-being, as many negative psychological effects of obesity are related to limitations in physical function of the individual [73,74]. At the same time, being overweight is a risk factor in the development of many diseases in humans and animals, and can exacerbate painful pre-existing conditions such as degenerative joint problems [49,71]. For cases where modification of body condition is desirable, it is therefore important to balance the psychological as well as physiological impacts of weight loss or gain to the animal.

To decrease an animal’s weight, it is common to restrict its caloric intake, and one of the ways this may be accomplished is reducing or eliminating additional feedings given during training. Although the food reward component of positive reinforcement training is a positive experience, particularly for highly food motivated individuals, the cognitive challenge and relationship building between a trainer and an animal can also have positive impacts on an animal’s welfare state [75]. If weight loss is an end goal, identifying alternatives to high calorie training rewards, or mixing a few high calorie rewards in with lower calorie alternatives (i.e., varying reward type/schedule [76]) can be useful for maintaining training opportunities for the animal while supporting reduced caloric intake. Conducting preference tests can help identify lower calorie food items an individual finds rewarding, and may aid in the substitution process. The assistance of a qualified nutritionist or veterinarian is advisable for identifying species appropriate food substitutions.

For animals requiring additional feedings to promote weight gain or maintenance, it is important to recognize supplemental feeding may not always be a positive experience, such as if the animal needs to be restrained for gavage feeding [77]. In such cases, providing animals with a reliable signal preceding the negative event can help mitigate the negative welfare impact [77]. Moderation, common sense, and creativity are key when balancing weight management goals with welfare opportunities for ageing animals. 

### 3.2. Skin and Feather or Coat Condition

Many mammals lose elasticity in their skin with age, due to a decline in collagen production. The production of sebum may also decline with age, leading to dry skin and associated symptoms such as itching [78,79]. Topical treatments may be challenging to administer daily in larger species, but such treatments could be candidate behaviors for positive reinforcement training. Chronic itching may be a type of negative welfare experience overlooked by classical definitions of welfare, as it does not necessarily fall under classical definitions of pain or suffering, though it is a form of discomfort [80]. Itching can lead to over grooming or self-mutilating behaviors in attempts to achieve relief [79,81,82]. These extreme behaviors suggest unrelieved itching is a negative experience and may create a feedback loop that generates further undesirable feelings. For example, excessive grooming may leave animals less able to thermoregulate due to hair loss [44,79], and self-mutilation may put animals at risk of infection due to wounds [82]. Thus, even in cases where no underlying medical cause of itching can be identified or itching is only related to skin condition, providing topical treatment of symptoms can help keep the animal comfortable, support positive welfare through training opportunities, and prevent future medical complications.

In cats and dogs, declines in overall coat quality with age are common, and in cats particularly this may be exacerbated by a decrease in normal grooming behaviors [44,83]. Changes in coat and hair growth have been observed in older horses as well [63]. In some animals, hair loss or thinning of coat may occur. Generally such changes may be largely superficial and have limited impact on the animal’s daily life, however should hair loss become extensive it may have a negative impact on an animal’s ability to thermoregulate [44,84]. In such cases, providing additional heat sources, shelters, or bedding can help the animal maintain an appropriate body temperature. Thermoregulation is important for physical health of animals, but also important for their welfare. Regularly experiencing inappropriate thermal extremes or conditions resulting in an inability to regulate body temperature is generally accepted to be an unpleasant experience [85,86,87] and careful consideration of changes to animals’ abilities to maintain proper body temperatures can help support comfort and welfare of older animals. Cold sensitivity may occur as a secondary effect of declining body condition and a reduction in movement in ageing animals. More ‘outside the box’ ideas to mitigate cold sensitivity or difficulty thermoregulating can include training animals to wear blankets when needed.

### 3.3. Dental Issues

Tooth wear is considered a natural age-related phenomenon in most (but not all) mammals [88]. Some degree of wearing is expected over time due to normal food processing, however abnormal tooth wear may be a significant cause of morbidity in a variety of species [89,90]. Dental pain may also contribute to digestive issues due to inability to properly chew food, or an overall decline in body condition associated with eating less [41,66]. Even without obvious symptoms, older animals may have extensive dental disease. A majority of geriatric domestic cats surveyed had significant plaque buildup at the time of death [57], and older domestic horses and sheep (*Ovis aries*) both have high rates of periodontal disease [65,91]. Retrospective assessments in jaguars (*Panthera onca*) and all species of extant bears in zoos indicate a high prevalence of dental disease, broken or missing teeth, and cavities [27,92]. Dental disease is common in aged great apes as well [93,94,95]. Bear skeletons from zoos showed signs of jaw bone abscesses potentially related to broken or worn teeth [27], and similar issues have been documented in living bears [96]. A study of captive and wild ring-tailed lemurs (*Lemur catta*) suggested less overall dental wear with age in captive lemurs compared to their wild counterparts [97]. This pattern may not hold true across all taxa, however, as several ungulate species show greater dental wear in captivity than in the wild [89,98], and captive gray wolves (*Canis lupus*) show similar patterns of dental wear to their wild counterparts [99]. As animal welfare must be considered at the individual level, assessing level of tooth wear between captive and wild animals is likely not informative for assessments of individual welfare, and animals will need to be assessed on a case-by-case basis for welfare concerns. At the same time, as dental health has significant impacts on overall health, understanding species level trends in dental health is important for providing proper veterinary care.

Dental wear in zoo animals can be related to both the dietary items fed, and method of feeding [89,90,98]. Both diet items and feeding mechanisms vary significantly among zoological institutions, suggesting dental wear patterns will likely vary among zoos as well as between wild and managed populations. As many zoo animals live longer than their wild counterparts and age is often a predictor of the degree of dental wear, animals in zoos may exhibit more extensive dental wear due to their longer life spans [1,88,99,100,101,102,103]. Animal managers should focus on maintaining good dental health or mitigating existing dental problems of individual animals to help them avoid dental related welfare concerns as they age.

In large species especially, dental issues are often uncovered while animals are under anesthesia. However, there are risks associated with using general anesthesia for older animals, and as such it may not always be feasible to conduct anesthetized exams for dental cleanings or treatment of dental disease once an animal reaches advanced age [104,105,106]. Including dental care and cleaning in regular health checkups and animal training plans throughout the life span of the animal is the first line of defense against dental disease in old age [65,70,96,107]. Lifelong dental care is a standard recommendation by veterinarians for companion animals and is increasingly a part of care plans for animals in zoos and aquariums [65,70,96,108]. As animals may not grow old at the same facility where they are born or raised, even institutions with established dental care protocols may end up caring for animals with significant dental issues [34]. Thus it is beneficial for facilities to have established management practices in place for animals with dental problems. Dental disease can be painful due to exposed tooth pulp or nerve involvement [96]. Untreated pain of dental disease is a significant welfare concern, especially as modern advances in veterinary dental care make suffering through dental pain unnecessary [27]. Pain due to dental problems can be associated with apparent changes in appetite or decreased food intake, being more selective about foods (e.g., avoiding hard to chew items), or digestive changes [43,48,64,65]. Providing animals with a varied diet with foods of different textures can provide opportunities to make choices in their daily life, and maintaining a varied diet into old age may give the animal some degree of control if or when they are experiencing dental issues. Consulting a nutritionist is important to ensure the variety of foods offered are nutritionally balanced, and for adjusting the amounts offered should the animal preferentially consume only a few items. A varied diet can serve the dual purpose of allowing animals to avoid foods which are painful to chew when they are experiencing dental pain and also supporting positive welfare by promoting decision making and agency. Dental or periodontal disease is also a risk factor for other medical complications, and proper attention to oral health can prevent potentially painful or fatal future health complications [34,65,70,96,107].

### 3.4. Degenerative Joint Disorders and ‘Lameness’

Degenerative joint problems have been documented both in vivo and post mortem in a large number of species (see Table 1). Many specific diagnoses are encompassed within the category of degenerative joint disease, however degenerative joint issues share many symptoms despite different underlying causes: limited range of motion, swelling of joints, changes to gait or posture, muscle weakness or instability, or changes in overall mobility (e.g., stiffness, difficulty laying down or standing up [25,49,51,70,71,72,109]). The widespread nature of degenerative joint changes related to age indicate this is a natural progression of ageing in vertebrates; however, differences in body structure and physiology cause species specific differences in which joints are most affected. For example, large felids are prone to osteoarthritic changes in elbow and stifle joints [106], domestic sheep may develop arthritic changes in the elbows or jaw [52,110], and non-human primates tend to develop arthritis in the stifle, hip, fingers, and spine [71]. While advances in veterinary radiographic imaging have greatly advanced the diagnosis of degenerative joint changes in living animals, it is important to note that pain or mobility limitations can still be significant, even if pathological changes are not visible on radiographic imaging [25,45]. Significant degenerative changes visible on radiographs may also be present apparently asymptomatically (e.g., no behavioral changes [45]). Moreover, changes to the biomechanics of an individual due to previous injury may present symptomatically in a manner similar to degenerative joint issues, but in fact be due to structural or mechanical changes that have healed without pain [111,112].

Veterinarians will be able to provide guidance on the best pain management treatments for animals suspected of having painful degenerative joint changes. As assessing pain levels is challenging in many exotic species, behavioral assessments before and after beginning treatments for pain can help identify relevant pain related behaviors [115]. Comparing behavior pre and post treatment for pain can also clarify whether changes in behavior (e.g., posture, gait, or activity levels) are related to pain [115]. When utilizing behavioral assessments for any purpose, consider the medications the individual is on, as side effects from medications may significantly alter animals’ behavioral patterns [116]. Although formal scientific studies of the use of non-traditional pain relief methods are lacking in most exotic species, physical therapy, chiropractic care, cold/heat therapy, liniments, farrier work, massage, cold laser treatment, acupuncture, and nutritional supplements may be additional inputs for supporting pain relief in ageing animals [117,118]. Whether non-traditional pain relief methods could be beneficial to an animal should be determined by attending veterinarians, and institutions should also consider whether funding agencies or governing bodies are supportive of alternative treatments. Treatments involving physical manipulation or exercises can be candidate behaviors for positive reinforcement training, which can provide opportunities for positive welfare experiences associated with learning and acquiring rewards while also supporting the animal’s pain relief, physical strength, and overall comfort [19,119,120].

Beyond pain related concerns of degenerative joint issues, routine assessment of space use by older animals can help identify if the animal has difficulty accessing spaces due to changes in mobility or strength. Modifying exhibits to provide shorter steps, less steep inclines, and larger or additional platforms can give the animal more options for using the entirety of its available space [49,58]. For ageing animals managed under protected contact, spaces may need to be modified to provide care staff or trainers safe access to the animal for treatments or therapy. Offering more bedding or raised bedding can also help animals achieve a greater degree of independence in the form of laying down or standing up more easily. Providing softer substrates can also afford relief to ageing joints, and there are many commercially available options for floor mats in barn stalls [49,67,70,106]. Assessment of how the animal responds to training or enrichment opportunities can also inform assessments of mobility and interest in the opportunity. If an animal readily engages with a trainer but no longer reliably offers a trained behavior she has known for years, this could suggest motivation to train that is tempered by physical pain or limitations (e.g., “lay down” is no longer possible for an animal on a training bench due to arthritis, but the animal could still lay down comfortably on the floor). Being able to manage one’s own processes (i.e., having the opportunity to make a choice or move around a space uninhibited) promotes independent decision making and supports positive welfare [8,121,122,123]. Mobility limitations associated with degenerative joint disease may restrict the ability of older animals to make choices about space use or exert control in their daily activities. Careful consideration of veterinary inputs and exhibit modifications can allow animals to continue to express independent decision making into old age.

## 4. Cognitive and Behavioral Changes

Behavioral assessments throughout animal’s lives can help clarify the normal behavior of individuals [124]. This in turn can provide valuable baseline information about how behavior is changing over time, and allow assessment of what factors are related to changes in animal behavior. Baseline behavioral observations can provide information as to whether the onset of behavioral changes are acute or developed slowly over time. Having longitudinal data on individuals can assist in determining whether changes in behavior reflect normal ageing (e.g., gradual changes), or are cause for concern.

### 4.1. Changes to Sleep Cycles

Many characteristics of animals’ circadian rhythms change as they age, and changes to various sleep characteristics have been noted in elderly humans and animals [125]. Aged animals may spend more time awake during their typical sleep period (e.g., diurnal animals awake at night or nocturnal animals awake during the day) or engage in shorter bouts of sleep at varied times throughout the day [2,26,35,125]. As restful sleep is important to physical and psychological well-being, providing flexibility in daily schedules for older animals is important [126]. For example, if an animal historically secured indoors in the morning begins sleeping through until early afternoon but is responsive to management activities later in the day, the best management option may be to shift the service schedule to accommodate the animal’s new circadian rhythms. This provides the animal with a degree of control over their own schedule. Ageing animals can be given an additional degree of control in their daily lives by giving them indoor/outdoor access throughout the day, and this may help animals self-regulate activity and stimulation levels as needed. Allowing older animals to set their own schedules may be more feasible for animals not kept in public areas, and maintaining areas where older animals can be ‘retired’ off exhibit may allow care staff to work with the changing circadian rhythms of older animals more readily. Such accommodation will require broader institutional support of welfare efforts for ageing animals, in terms of staffing schedules and facilities.

### 4.2. Changes to Activity Levels

While older animals may need to sleep less, declines in overall activity levels are also commonly associated with ageing across many species (Table 2 [60,61]). As with many aspects of ageing, a slow decrease in activity over time can be a normal sign of getting older; however, sudden or excessive declines in activity may indicate underlying medical issues, psychological issues, changes in physical ability of the animal, or limited opportunities for the animal to engage with the environment [49,52,106]. Furthermore, low activity levels are not inevitable for ageing animals [42,48], and animals exhibiting excessive lethargy or apathy should be assessed for underlying medical or psychological issues at any life stage [127]. Behavioral monitoring throughout animals’ lives can clarify what a ‘normal’ activity level is for an individual, and help determine whether changes in activity levels occur gradually over time or suddenly. Beginning behavioral assessments before concerns arise can provide valuable baseline information about an animal’s ‘normal’ for care staff, veterinarians, and animal managers when changes occur [124].

### 4.3. Changes in Social Interactions

Age-related changes in animal’s physical abilities and behaviors may also impact their interactions within social groups. Ageing animals can lose social standing within a group if they are no longer able to actively engage in social interactions with other individuals [32]. They may also become more aggressive [35]. It is also possible that social interactions can become more important to animals as they age because social interactions can be a significant source of comfort for some species [129,130,131]. Ageing animals may also commonly become less interested in joining in with a group [26,45]. Similar changes are possible related to an animal’s interest in interacting with caretakers—the animal may become less interested or more interested in interacting with caretakers as it ages [32,35,44]. Again, small shifts or gradual changes in social engagement can suggest a normal course of ageing, but sudden isolation or aggression can indicate a more serious underlying medical issue (e.g., undiagnosed pain or neurological changes [132]).

For animals managed in social groups or herds, providing additional choice and control for old animals within the bounds of normal social interactions can help maintain positive welfare throughout their lives. Subdividing available spaces can provide more ‘private’ areas where the animal can go to avoid unwanted social interactions. Caretakers may also need to more extensively manage social sub-groups in order to provide older animals companionship with less or no social conflict [133,134,135]. In interactions between the animal and caretakers, the animal should always have the choice available to not engage with keeper staff, with the caveat that choosing to not engage is different than being physically or mentally unable to engage (e.g., apathy or inability to stand compared to relaxed and responsive but choosing not to participate [45,127]). Furthermore, animals who have preferences for specific care staff may become more rigid in their preferences with age, and animals who previously did not appear to have preferences may develop them. Older animals may also become less tolerant of strangers or new staff due to physical pain or cognitive changes. Careful monitoring of animals’ behavioral responses to various situations may help managers decide a course of action that reflects animals’ desires and simultaneously supports successful husbandry.

### 4.4. Confusion, Disorientation, and Slower Learning

Brief episodes that appear to be slips in cognitive function are a normal part of ageing in humans, and have been documented in several species [26,35,136]. Due to the challenge of designing and deploying cognitive assessments for animals, most evidence for cognitive decline with age in animals comes from animal models of human ageing such as rhesus macaques (*Macaca mulatta*), or from domesticated species [26,136]. Thus, it is probable more species than have been formally assessed experience age-related changes to cognitive ability. Memory lapses, or momentary confusion or disorientation may become more frequent with age. These symptoms may impact the animal’s ability to navigate well-known spaces, recognize social companions or keepers, and their performance with training or enrichment activities [26,44,136]. The response time of older animals to cognitively challenging tasks may become longer and learning new behaviors or mastering new tasks may take more time as well [136]. As cognitive research in zoos and aquariums becomes more common, it may become feasible to empirically assess cognitive changes related to age in a wider variety of species [137].

The behavioral symptoms of changes in cognition may present similarly to (or alongside) sensory declines, and it is useful to rule out changes in sight or hearing before assuming the animal is experiencing age-related cognitive issues. Once underlying veterinary issues are ruled out, animals experiencing cognitive changes, memory lapses or disorientation should be managed on a case-by-case basis. Caretakers must be more patient with animals experiencing this kind of behavioral change, as regular husbandry activities may take longer than expected [26,31,57]. Encouraging care staff to recognize the animal is not just being ‘difficult’ or ‘bad’ when an elderly animal has difficulty responding can help mitigate negative feedback to the animal from caretakers. As the human-animal relationship can be a source of positive welfare experiences for animals [138,139,140,141], maintaining this positive relationship becomes especially important for elderly animals having difficulties with daily activities. Maintaining consistency in daily patterns can help animals experiencing this issue cope, as short-term memory is often impacted by cognitive decline before long-term memory [128]. For instance, daily routines the animal has done for years may remain consistent, whereas recently learned behaviors may become less consistent. Simplified training plans, with a limited number of trainers or simpler cues may benefit animals showing signs of cognitive decline.

Many animals show a decreased ability to learn new things with age, and this highlights the importance of lifelong planning for behavioral management and training. Elderly animals may not be able to quickly learn new behaviors, even when the behaviors would be beneficial to their health and management. Lifetime training plans are generally not the norm but may be useful to help prepare for age-related changes. Such plans would include specific focus on training needs associated with particular ‘life stages’. They might focus on timing the training of behaviors such that they are reliable prior to their expected need for a given life stage. For example, training an animal to sit for cardiac ultrasound assessment can begin in mid-adulthood, potentially before the ultrasound results are medically relevant but also before the animal experiences medical or behavioral declines related to old age.

### 4.5. Abnormal Repetitive Behaviors

The development of abnormal repetitive behaviors at any age should prompt an assessment of the environment, management, and physiological state of the animal exhibiting the behavior, as such behaviors can be indicative of underlying medical issues or negative welfare states [142,143]. When an animal who has not previously exhibited abnormal repetitive behaviors develops one suddenly, this can indicate acute pain, discomfort, neurological changes, or changes to their psychological state. Rapid onset of abnormal repetitive behaviors has been observed in animals with advanced neurological changes or ischemic events [31,144]. As we recognize distress is incompatible with positive welfare, we should also recognize that an animal exhibiting a sudden abnormal repetitive behavior in an environment that has not recently changed may still require additional interventions to be able to achieve positive welfare. If the behavior does not have a diagnosable physiological component (e.g., rapid neurological changes due to tumors or illness), conducting a complete assessment of the environment and overall welfare state of the animal can be useful, as animals living in well-provisioned environments may still experience psychological distress contributing to the development of the behavior [6].

### 4.6. Sensory Decline

Sensory decline is common in older vertebrates. For example, many species will experience changes to the lens of the eye with age. Cataracts are common in ageing animals, and can range in severity from asymptomatic to severely limiting animal’s eyesight [145,146]. Similarly, hearing may decline as animal’s age, and the ability to hear high frequencies declines sooner than low frequencies in most cases [44]. Sensory declines are often related to physiological changes, but may be first suspected or detected due to changes in behavior. Human caretakers likely most often interact with animals on a visual or auditory basis, as these are the senses humans tend to rely on [147]. Managers and caretakers should consider the possibility of changes in the animals’ senses besides hearing and sight, as decreased olfactory sensitivity or decreased sensitivity of other dominant senses may impact the animal’s behaviors and demeanor in unknown ways. Being sensitive to the possibility of changing umwelts of ageing animals may necessitate creative problem solving, particularly for animals experiencing declines in multiple sensory systems at once.

While most animals experiencing changes to their sensory abilities can adapt to their daily lives with few or limited impacts on their overall welfare state, caretakers can help mitigate the potential negative welfare impacts of sensory decline. Startling or frightening animals is a negative experience [148] and as such caretakers should consider animals’ limitations when approaching them or arranging the animal’s yard. For instance, visually impaired animals may need to have audio cues indicating human presence or location, whereas hearing impaired animals may benefit from having lights flashed or other visual cues when a human enters their space. Training and enrichment activities should be modified to accommodate the animal’s sensory abilities—there are few circumstances where an animal’s physical limitations justify limiting their enrichment or training opportunities. Animals with physical or sensory limitations are often more in need of training as they may not interact with enrichments provided to younger or more able-bodied counterparts. Blind or vision limited animals may not engage with object enrichments if they cannot find them for example, and thus they can miss out on enrichment available to other animals. However, the same vision impaired individual can experience positive welfare opportunities by engaging in training sessions with caretakers, assuming the training uses audio cues the animal can perceive.

### 4.7. Senile Plaques—A Physiological Basis of Ageing Brains?

The cognitive and behavioral changes observed in ageing animals can be attributed to a number of physiological and neurological changes [36]. One commonly reported neurological change observed in many ageing vertebrates is the accumulation of amyloid protein plaques in the brain (see Table 3). The presence of these plaques is associated with the symptoms and cognitive declines occurring in individuals with Alzheimer’s disease in humans [149]. Although similar behavioral changes have been observed in great apes, the association between cognitive declines and concentrations of amyloid plaques is inconclusive in other animals [36,150,151,152]. This may be partly due to the post-mortem identification of plaque concentration during necropsy, coupled with a lack of longitudinal behavioral data for most animals throughout their lives [31,153,154]. The development of these protein deposits in the brain may represent a taxonomically conserved physiological basis of neurological ageing in vertebrates [155,156,157,158]. As this type of neurological change is not possible to diagnose while animals are still alive, animal managers and caretakers are more likely to encounter changes in animal cognition, motivation, or behavior without knowing the etiology of the changes. Nevertheless, knowing that such changes can have a physiological basis may help caretakers be more understanding toward changes in behavior, thereby supporting a positive human–animal relationship in daily husbandry and care [141].

### 4.8. Managing Behavioral Changes

Depending on the specific behavioral issue, managing behavioral and cognitive changes in elderly animals can generally occur without knowing the specific underlying cause. Moreover, gradual changes may be a normal part of the ageing process [26,35]. Distinguishing between normal age-related cognitive decline and early signs of degenerative neurological changes is difficult in humans even with the battery of assessments at the disposal of medical professionals [162]. Thorough and ongoing behavioral assessments of ageing animals can be useful to this end. 

Monitoring the behavior of ageing animals during cognitively challenging activities such as training and assessing changes in performance over time can help clarify whether animals are experiencing normal or abnormal age-related changes. In humans, degenerative cognitive disorders can cause confusion, resulting in frustration or inappropriate aggression [163,164]. For animals, frustration and confusion may limit their ability to experience positive welfare benefits associated with positive reinforcement training or caretaker interactions [26,35]. Identifying animals potentially experiencing cognitive declines can help trainers adjust training plans and managers adjust husbandry to prevent frustration, aggression, or other negative experiences.

## 5. Neoplasia, Cardiovascular Disease, and Other Age-Related Physiological Issues

A large body of veterinary research in zoos supports age as a risk factor for animals developing a variety of diseases. Specifically, the risk of neoplasia, cardiovascular disease, and metabolic disease increases with age many species [29,34,45,95,144,165]. Differences in physiology, natural history, and genetics cause some species to have higher risks of developing specific age-related health issues than others [29,45,106,166]. Understanding differences in species level risk is important for providing proper veterinary care to all animals, however the welfare implications of such health problems are less clear. If an age-related disease causes pain or suffering, detracts from an animal’s ability to behave normally, exert agency, acquire rewards, or have other positive welfare experiences, the disease becomes a welfare issue. If the disease can be treated and upon treatment the animal is still able to have positive welfare experiences, or if the disease is diagnosed in an animal exhibiting no signs of compromised welfare, treating the disorder medically is appropriate. Animals experiencing health problems should be closely monitored for changes in behavior and welfare status at any age, however consistent monitoring is critical for older animals where subtle pain behaviors or changes to activity may be more readily attributed to their age. The development of welfare assessments that can be used for life-long welfare studies of individual animals will be important for clarifying the interaction between animals’ physical health and welfare states. 

## 6. Welfare Considerations in End-of-Life Decision Making and Humane Euthanasia

No discussion of ageing animal care would be complete without considering end-of-life decision making and humane euthanasia. Much has been written on the ethical considerations involved in deciding to humanely euthanize pets or other domestic animals, and many of the same principles must be considered in end-of-life decision making for animals in zoos or aquariums. Specifically, animal well-being should be the highest priority when deciding if or when to humanely euthanize any animal [2,57]. Although this standard seems easy to put forward, objectively deciding when humane euthanasia is appropriate is a difficult decision. In animal care institutions where such decisions must be defensible to many stakeholders, end-of-life decision making takes on additional levels of complexity. Drawing on the domestic animal literature has limitations for zoos or aquariums, as owners of domestic animals may be able to more easily handle animals for treatments, or may prolong the animal’s life due to their own emotional distress over the loss of a pet [2]. Production animal or livestock research provides limited guidance as well, as many animals raised for food production usually do not live to see old age [167,168]. Thus, the issue of if or when to humanely euthanize an aged animal in a zoo or aquarium setting is an ethical dilemma faced by modern zoos that has limited parallels to other fields of animal care [169].

In zoos and aquariums, veterinarians are responsible for identifying cases where humane euthanasia is medically appropriate due to poor prognoses or to end untreatable pain and suffering of an individual. Making end-of-life decisions for animals experiencing slower or more gradual declines in welfare may be less clear cut, and may require input from primary care staff, managers, veterinarians, and behavior staff. The top priority of institutions should be providing animals with a high quality of life for as long as possible. At the same time, prolonging the length of an animal’s life to the point where it is no longer able to experience positive welfare must be avoided. Often though, animals remain able to have more positive welfare experiences than negative ones in spite of declining health or physical ability, and providing as much time as possible to experience good days is desirable [170]. Aged animals with some degree of debilitation can still have a positive quality of life, although physical limitations may lead to concerns over keeping aged animals in view of the public. In situations where concerns over public perception of ageing animals arise, it can be useful to improve signage or provide interpretation about the animal’s age or condition for the public. It may be beneficial to move aged animals into off-exhibit spaces to allow the institution to focus on maximizing positive welfare opportunities even at the end of an animal’s life.

Formal quality of life assessment methods have been proposed for small animals or pets under hospice care [171], and other methods have been proposed for use in zoological institutions [45,170]. Adopting similar methods may be helpful when assessing cases of declining welfare. The development of comprehensive quality of life assessments for ageing animals is an important step in the advancement of the care and welfare of aged animals in zoos and aquariums, and should be an area of future research. 

## 7. Future Directions and Institutional Considerations

### 7.1. Staffing and Expertise

Caring for aged animals requires highly individualized care across many different areas of expertise. For any single elderly animal, an institution may find itself in need of input from nutritionists, general veterinary practitioners, veterinary specialists, behaviorists, trainers, exhibit designers, or construction experts. While most institutions have a number of these experts on staff, few will have all of the expertise needed for every situation. Thus, it is important to maintain open communication with outside consultants when needed, and to provide training opportunities for staff to help develop the institution’s working knowledge of how to manage the later life needs of animals across taxa. As ageing animals may require additional time dedicated to them for daily husbandry, enrichment, or training, institutions may need to consider adding personnel and resources as part of their approach to caring for aged animals. 

### 7.2. Taxon Specific Concerns

Although there are many commonalities in the ageing process across taxonomic groups, improvements to husbandry, management, and veterinary care of individual species will require studies across zoo and aquarium populations. Such studies will not be possible without multi-institutional collaborations that consider age-related changes in specific species. Increasing numbers of retrospective analyses of morbidity and mortality across institutions are being published [29,45,49,92,146]; however, these are often based on reviews of veterinary records. While this information is important for informing veterinary care and understanding of species specific disease risks, medical records often do not include information about husbandry, behavior, or management practices the animal experienced. Improved information sharing among zoos regarding husbandry, management, and veterinary records would support more holistic assessments of species specific age-related health and welfare concerns [172,173]. 

While reptiles do not appear frequently in animal welfare literature, increasing evidence suggests they also experience the welfare benefits associated with enrichment and more complex environments [174,175,176,177]. At the same time, few studies have addressed behavioral or physiological changes associated with ageing in reptiles in captivity or in the wild (but see [178,179]). Given the long life spans of many reptilian species and the overall lack of research into the welfare experiences of reptiles, this is an area where more work is needed to improve management and care of ageing reptiles. Nevertheless, given the apparent generalities of age-related issues that develop in other taxa, perhaps it is reasonable to assume that similar concerns may arise for reptiles—but perhaps over a much longer period of time.

### 7.3. Lifelong Care and Training

Caring for animals in zoological institutions is a cradle to grave endeavor—most animals born and raised in a zoo will live out their lives in one. Planning ahead for different life stages is therefore critical for the welfare of all animals living in zoos. In the same way younger animals can be trained to participate in their own medical care voluntarily, older animals can become active participants in their own therapy and treatments as well. Animals at any life stage will experience the benefits of added choice and control over their daily lives, and the medical care that can be given voluntarily can provide better outcomes than higher stress alternatives [180]. At the same time, animals who have never trained a medical behavior before may not be able to quickly learn to sit for a blood draw or injection once they fall ill or experience age-related declines. Beginning training efforts for medical behaviors before they are necessary requires lifelong planning for animals under human care.

## 8. Conclusions

Ageing animals may be uniquely vulnerable to a number of negative welfare experiences when compared to younger animals, including painful physical changes or medical conditions associated with ageing, frustration due to changing physical abilities, social difficulties, or cognitive changes. Individualized management can help mitigate the effects associated with physiological and cognitive aspects of ageing, and thoughtful applications of age-appropriate enrichment and positive-reinforcement training can help keep animals physically and psychologically fit throughout their lives. Providing opportunities for choice and exerting control in daily life—from food to social groupings to exhibit use—is also critical to supporting positive welfare of aged animals under human care. As many animals hide pain or exhibit non-descript pain behaviors [181], providing choices can help animals manage their own experience in a way that maximizes their comfort and opportunities for positive welfare. Positive reinforcement training is a useful method for engaging the animal in its daily care, and consistent assessment of training performance and motivation can help identify age-related changes in physical and cognitive abilities. Training also provides opportunities for animals to solve problems and acquire rewards, and fosters a positive human animal relationship between animals and their caretakers, all of which can support positive welfare in animals [75,141] Behavioral monitoring can help identify areas where individual animals may need additional interventions to support their opportunities for positive well-being.

Supporting positive welfare at all life stages is critical to the mission of accredited zoos and aquariums, and by understanding the similarities and potential management solutions for common challenges of ageing across species, we can help support the positive welfare of animals under human care throughout their lives.

## Figures and Tables

**Table 1:** Common physical changes described in ageing animals of different species.

Physical Change	Species	References
Dental disease	Domestic cats, domestic sheep, horses, gorillas (*Gorilla gorilla)*, chimpanzees (*Pan troglodytes)*, bonobos (*Pan paniscus*), orangutans (*Pongo pygmaeus*), ring-tailed lemurs, brown bears (*Ursus arctos*), jaguars, blackbuck (*Antilope cervicapra*), axis deer (*Axis axis*), American bison (*Bison bison*), nilgai (*Buselaphus tragelaphus*), domestic goats (*Capra hircus*), Eld’s deer (*Cervus eldi*), black wildebeest (*Connochaetes gnou*), goitered gazelles (*Gazella subgutturosa*), oryx (*Oryx leucoryx*), reindeer (*Rangifer tarandus*), greater kudu (*Tragelaphus strepsiceros*), lesser mouse deer (*Tragulus javanicus*)	[44,45,66,70,91,92,93,94,95,96,106,107]
Degenerative joint disease	Domestic dogs, domestic cats, sheep, horses, gorillas, chimpanzees, bonobos, orangutans, rhesus macaques, brown bears, polar bears (*Ursus maritimus*), sun bears (*Helarctos malayanus*), spectacled bears (*Tremarctos ornatus*), tigers (*Panthera tigris*), leopards (*Panthera pardus*), snow leopards (*Panthera uncia*), African lions (*Panthera leo*), ocelots (*Leopardus pardalis*), mountain lions (*Puma concolor*), jaguars, cheetahs (*Acinonyx jubatus*), wolves (*Canis lupus*), African elephants (*Loxondata africana africana*), Indian rhinoceroses (*Rhinoceros unicornis*), black rhinoceroses (*Diceros bicornis*), sable antelope (*Hippotragus niger*), greater kudu, koalas (*Phascolarctos cinereus*)	[25,44,45,49,51,52,57,63,67,70,71,72,92,94,95,106,109]
Changes to body condition or composition	Domestic dogs, domestic cats, humans, domestic sheep, horses, chimpanzees, rhesus macaques, squirrel monkeys (*Saimiri sciureus*), cynomolgus monkeys (*Macaca fascicularis*), African lions, tigers, leopards, snow leopards, jaguars	[40,42,44,49,52,57,92,94,113,114]
Changes to skin or fur	Domestic dogs, domestic cats, horses, rhesus macaques, jaguar	[44,63,66,67,78,83,92]

**Table 2:** Common behavioral changes described in ageing animals of different species.

Behavioral Change	Species	References
Changes in sleep patterns	Domestic dogs, domestic cats, humans, mice (*Mus musculus*), wolverines (*Gulo gulo*)	[26,31,44,125]
Changes in social interactionswith caretakers or other animals	Domestic dogs, domestic cats, gorillas, wooly monkeys (*Lagothrix lagotricha*), green monkeys (*Cercopithecus aethiops*), spider monkeys (*Ateles geoffroyi* spp.), crab–eating macaques (*Macaca fascicularis*), African lions, tigers, leopards, snow leopards, jaguars, wolves, okapis (*Okapia johnstoni*), musk oxen (*Ovibos muschatus*), Masai giraffe (*Giraffa camelopardalis tippelskirchi*), sable antelope, greater kudu, Bactrian camels (*Camelus bactrianus*), Grevy’s zebras (*Equus grevyi*), Chapman’s zebras (*Equus quagga chapmani*), African elephants, Indian rhinoceroses, black rhinoceroses, Brazilian tapirs, wolverines	[25,26,31,32,35,44,45,128]
Decline in overall activity level	Domestic dogs, domestic cats, domestic sheep, rhesus macaques, silvered-leaf monkey, lion-tailed monkey, moor macaque, wooly monkeys, green monkeys, spider monkeys, crab eating macaques, African lions, tigers, leopards, snow leopards, jaguars, brown bears, polar bears, sun bears, spectacled bears, wolves, okapis, musk oxen, Masai giraffe, sable antelope, greater kudu, Bactrian camels, Grevy’s zebras, Chapman’s zebras, Indian rhinoceroses, black rhinoceroses, Brazilian tapirs, African elephants, mice, rats (*Rattus norvegicus*), Mongolian gerbil (*Meriones unguiculatus*), fruit flies (*Drosophila melanogaster*)	[26,44,49,52,56,60,61]
Changes in grooming/bathing behaviors	Domestic cats, brown bears, polar bears, sun bears, spectacled bears, jaguars	[44,45,92]
Decrease in appetite	Domestic dogs, domestic cats, domestic sheep, wooly monkey, green monkey, spider monkey, crab-eating macaque, African lions, tigers, leopards, snow leopards, jaguars, wolves, okapis, musk oxen, Masai giraffe, sable antelope, greater kudu, Bactrian camels, Grevy’s zebras, Chapman’s zebras, Indian rhinoceroses, black rhinoceroses, Brazilian tapirs, African elephant	[26,35,44,45,52,92,128]

**Table 3:** Species exhibiting amyloid protein deposits (senile plaques) in the brains of ageing individuals.

Species	References
Domestic dogs, domestic sheep, domestic goats, chimpanzees, orangutan, gorilla, squirrel monkey, Campbell’s guenons (*Cercopithecus mona campbelli*), rhesus macaques, baboons (*Papio* spp.), mouse lemurs (*Microcebus murinus)*, black bears (*Ursus americanus*), spectacled bears, polar bears, guanacos (*Lama guanicoe*), reindeer, bison, rabbits (*Oryctolagus cuniculus*), wolverines, California sea lions (*Zalophus californianus*), great spotted woodpeckers (*Picoides major*), kokanee salmon (*Oncorhynchus nerka kennerlyi*)	[31,128,151,152,153,154,155,156,157,158,159,160,161]
